# Anatomy, Imaging, and Surgical Treatment of Thoracic Lymphadenopathies in Advanced Epithelial Ovarian Cancer

**DOI:** 10.3390/cancers16233985

**Published:** 2024-11-27

**Authors:** Stefania Rizzo, Maria Luisa Gasparri, Lucia Manganaro, Filippo Del Grande, Andrea Papadia, Francesco Petrella

**Affiliations:** 1 Clinica di Radiologia EOC, Istituto Imaging della Svizzera Italiana (IIMSI), Via Tesserete 46, 6900 Lugano, Switzerland; stefaniamariarita.rizzo@eoc.ch (S.R.); filippo.delgrande@eoc.ch (F.D.G.); 2 Facoltà di Scienze Biomediche, Università della Svizzera Italiana (USI), Via Giuseppe Buffi 13, 6900 Lugano, Switzerland; marialuisa.gasparri@eoc.ch (M.L.G.); andrea.papadia@eoc.ch (A.P.); 3 Department of Gynecology and Obstetrics, Ente Ospedaliero Cantonale (EOC), 6900 Lugano, Switzerland; 4 Department of Radiological, Oncological and Pathological Sciences, Sapienza University of Rome, Policlinico Umberto I, Viale del Policlinico 155, 00161 Rome, Italy; lucia.manganaro@uniroma1.it; 5 Department of Thoracic Surgery, Fondazione IRCCS San Gerardo dei Tintori, 20900 Monza, Italy

**Keywords:** ovarian cancer, lymphadenopathies, anatomy, imaging, surgery

## Abstract

Ovarian cancer typically presents at advanced stages, with prognosis heavily influenced by the presence of residual disease following cytoreductive surgery. The role of resecting enlarged extra-abdominal lymph nodes during cytoreductive procedures remains contentious. These enlarged lymph nodes are commonly identified through high-resolution imaging techniques such as ultrasound (US), computed tomography (CT), magnetic resonance imaging (MRI), and positron emission tomography–computed tomography (PET-CT). A comprehensive understanding of the relevant anatomy, imaging modalities, and surgical techniques for addressing lymphadenopathy in regions such as the supraclavicular fossa, axillae, mediastinum, and pericardiophrenic fat is crucial in determining the feasibility of surgical intervention. An appropriate evaluation of these factors is essential to optimize debulking, which is recognized as the most significant prognostic determinant in patients with ovarian cancer. This review underscores the importance of multidisciplinary approaches in managing advanced ovarian cancer with extra-abdominal lymph node involvement to enhance patient outcomes.

## Simple Summary

Ovarian cancer is often diagnosed at advanced stages, making treatment more challenging. One key factor that affects survival is the amount of cancer left after surgery. There is ongoing debate about whether removing enlarged lymph nodes outside the abdomen during surgery improves outcomes. These lymph nodes can be seen through advanced imaging techniques so that the best surgical methods can be adopted. This approach aims to ensure that as much of the cancer is removed as possible, which is crucial for improving patient prognosis. This review emphasizes the importance of careful knowledge of focused anatomy, imaging, and surgical treatment for advanced ovarian cancer patients with extra-abdominal lymphadenopathies.

## 1. Introduction

Ovarian cancer (OC) is the most common cause of death due to gynecologic malignancies, with 19.680 new cases and 12.740 estimated deaths in the US in 2024 [1]. Despite considerable progress in the medical treatment of OC in the past years, the cornerstone of the management of OC remains to be upfront surgery (primary debulking surgery) and a platinum-based combination chemotherapy. In case complete cytoreduction is not amenable upfront, neo-adjuvant chemotherapy can precede surgery [2]. The aim of surgery is complete (or at least maximal) cytoreduction. Indeed, it has been demonstrated that each 10% increase in maximal cytoreduction is associated with a 5.5% increase in median survival time [3]. However, only 12.3% of patients with FIGO (International Federation of Gynecology and Obstetrics) stage IV, referring to patients with distant metastases excluding peritoneal metastases [4], achieve complete cytoreduction [5]. A study including 2772 patients suggested that extraperitoneal metastases should not automatically preclude primary cytoreductive surgery consideration in suitable patients [6]. A study including more than 33,000 women demonstrated that OC patients with isolated distant node metastases had better survival compared with patients with stage IV disease based on other metastases sites and comparable survival compared to patients with stage IIIC disease [7].

There has been discussion regarding the extent of surgery, particularly concerning the role of pelvic and/or para-aortic lymphadenectomy. A randomized prospective trial found that systematic lymphadenectomy did not improve recurrence-free survival or overall survival (OS) but was associated with a higher rate of postoperative complications in primary cytoreductive surgery [8]. Notably, patients with visibly involved nodes were excluded from the trial and underwent systematic lymphadenectomy. However, more than half of those who had this procedure still showed positive nodes in their final pathology results. The specifics of preoperative imaging techniques were not thoroughly described; these included ultrasound (US), computed tomography (CT), or magnetic resonance imaging (MRI) for abdominal and pelvic assessments, as well as x-ray, CT, or MRI for chest evaluations [8]. To evaluate the lymph nodes, the retroperitoneal space was accessed from the inguinal ligament to the renal vein.

If on one side, retroperitoneal lymphadenectomy is not always performed; on the other, the presence of extra-abdominal enlarged lymph nodes indicates a FIGO stage IV disease, thus suggesting the option to let the patient undergo neo-adjuvant chemotherapy followed by interval debulking surgery and adjuvant chemotherapy.

However, with the goal of complete cytoreduction in mind, in recent years, gynecologic surgeons have become more and more committed towards finalizing a complete resection of all visible diseases [9], thus increasing the role of complex surgical procedures [10,11] and making the knowledge of extra-peritoneal anatomy increasingly important [12], not only for surgeons but also for radiologists involved in multidisciplinary meetings [13].

The pre-operative evaluation of OC patients include CT, PET-CT, and MRI [14]. According to the European Society of Urogenital Radiology (ESUR) guidelines, CT is the imaging modality of choice when OC is suspected or diagnosed [15]. The evaluation of lymph nodes at imaging has been debated over for a long time because there is still no clear consensus on the morphologic criteria that should be used, although a short axis <10 mm is usually accepted [16]. New classification systems and modalities have been introduced and will be discussed for each thoracic site considered in this review.

**Figure 1:**
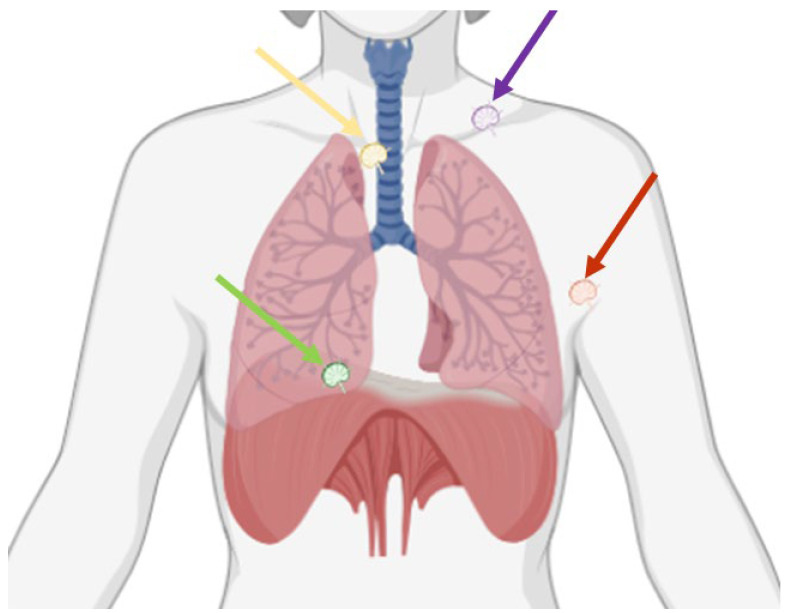
Possible thoracic sites of lymphadenopathies in ovarian cancer are the supraclavicular fossa (purple arrow), the axilla (red arrow), the mediastinum (yellow arrow), and the pericardiophrenic fat (green arrow).

Indeed, close multidisciplinary collaborations and advancements in imaging and surgical techniques may allow selected, previously deemed inoperable, stage IV disease patients to be considered for complete cytoreductive surgery. Therefore, the goal of this paper is to review the anatomy, imaging, and surgical techniques of extra-abdominal lymphadenopathies in OC patients (Figure 1), with a specific focus on the diagnosis and surgical resection of lymphadenopathies located in the supraclavicular fossa, in the axilla, in the mediastinum, and in the pericardiophrenic fat (Table 1).

## 2. Supraclavicular Fossa

### 2.1. Anatomy

The supraclavicular fossa, also known as the supraclavicular region, is delineated superiorly by the base of the neck, inferiorly by the clavicle, laterally by the shoulder, and medially by the sternocleidomastoid muscle. This region contains the subclavian artery and vein as well as the brachial plexus and lymph nodes [18]. The primary artery present is the subclavian artery, which is divided into three distinct segments by the anterior scalene muscle. The third segment—located distal to the anterior scalene—runs through the supraclavicular fossa. Additionally, the transverse cervical and suprascapular arteries traverse this fossa after branching from its first segment. The supraclavicular fossa contains the external and internal jugular veins.

The primary neural structures within the supraclavicular fossa are divided into superficial and deep nerves by the fascial layers of the neck. Superficially, the vagus runs within the carotid sheath. Deep within this fascial layer lies the phrenic nerve (C3-C5), which innervates the diaphragm. The phrenic nerve travels along the anterior scalene muscles toward the diaphragm. Also present in the supraclavicular fossa is the brachial plexus (C5-T1). The supraclavicular fossa houses various lymphatic structures that differ by laterality. On the left side, the thoracic duct serves as the principal structure, collecting lymph from the majority of the body before draining into the origin of the brachiocephalic vein. On the right side, the right lymphatic duct drains lymph from the right thorax, right upper extremity, and the right side of the head and neck, emptying into either the subclavian vein or the right internal jugular vein. These lymph nodes represent a distant spread location in OC.

### 2.2. Imaging

The supraclavicular fossa is usually included in a standard pre-operative thorax, abdomen, and pelvis CT scan extended from the lung apices to the pubic symphysis. The ongoing debate about the imaging characteristics of pathologic lymph nodes involves, in general, all lymph nodes, including the ones in the supraclavicular fossa. Eventually, the size cutoff depends on the desired sensitivity and specificity [17]. Although the nodal size is not considered an absolute criterion to indicate metastatic lymph nodes, a criterion widely accepted and, for this reason, included in the Response Evaluation Criteria and the Solid Tumors (RECIST) criteria, is a short-axis diameter <10 mm [19]. The recently introduced Node-RADS classification divides nodal sizes into three categories: “normal”, indicating a general short axis <10 mm; “enlarged”, indicating either lymph nodes larger than normal but not bulk, or an increase equal to or higher than 2 mm compared to prior imaging; “bulk”, indicating lymph nodes with any axis higher or equal to 30 mm [17]. The interesting adjunct of the Node-RADS classification is a score attributed to the node configuration, as second-level evaluation. Mainly, the configuration is evaluated according to three categories, including “texture”(homogeneous, heterogeneous, with fat necrosis, with gross necrosis or any necrosis), “border” (smooth, irregular or ill-defined), and “shape” (any shape with preserved fatty hilum, kidney-bean-like or oval without fatty hilum, spherical without fatty hilum). The node-RADS classification can be used with both CT and MRI. For application on PET-CT, it is considered applicable to baseline staging and to the evaluation of the response to therapy [17].

MRI is increasingly used prior to surgery for the evaluation of different solid cancers [20,21], including ovarian cancer, with the aim of evaluating the disease extension and identification of tumor deposits that may hinder optimal debulking, according to the center experience, available technology, and preference [22,23,24]. MRI may include only abdomen and pelvis, completed by a thorax CT, or performed as a whole-body technique. Whole-body MRI may be considered for a site-based analysis of sub-centimetric lesions, where DWI sequences, combined with morphological sequences, may outperform CT [22] and PET-CT [23]. However, this technique is less available, more expensive, longer, and thus harder for patients. Furthermore, in the specific setting of lymph node evaluation, no clear advantage has been demonstrated compared to other techniques, such as PET-CT, that remains the main imaging technology capable of displaying biomolecular metabolism, receptor activity, and neurotransmitter activity in vivo [25].

A meta-analysis including data from 882 patients with ovarian cancer showed that PET-CT was a more accurate modality for detecting metastatic lymph nodes [25]. However, FDG uptake may not be able to clearly discriminate inflammatory processes from cancer cells, and FDG uptake may not be increased in low-grade tumors [24,25,26,27]. Furthermore, clear conclusions cannot be drawn because of marked asymmetry among publications, suggesting that studies with favorable results are more likely to be submitted and published [25].

### 2.3. Surgical Resection

The supraclavicular lymph nodes are routinely approached by scalene lymph node biopsy as first described by Daniel in 1949 [28]. It can be performed under local or general anesthesia, depending on the volume and depth of lymph nodes to be approached as well as from the aim of the biopsy (incisional versus excisional biopsy).

The skin incision is performed over the clavicle and laterally to the sternocleidomastoid muscle. The clavicular aspect of this muscle is easily dissected, and the whole muscle is then medially retracted. This exposure allows a proper and safe visualization of the surface under the anterior scalenus muscle, containing a fat pad with multiple lymph nodes, which can be safely resected. There is usually no need for drainage, and cosmetic results can be acceptable thanks to an intradermal suture. Care must be taken not to injure the phrenic nerve, which, in the neck, lies on the anterior surface of the anterior scalene muscle; lymphatic leak can be properly prevented by clipping small lymphatic ducts of the district.

## 3. Axilla

### 3.1. Anatomy

The axilla is an anatomical space situated between the upper limb and the thorax, containing neurovascular and lymphatic structures. It is pyramidal-shaped, with its apex directed superiorly toward the base of the neck. The anterior border is delimited by the pectoralis minor, the clavi-pectoral fascia, the clavicle bone, and pectoralis major, which, together, form the anterior axillary fold. The serratus anterior muscle delimits the medial border, which overlays the chest wall up to the fourth rib. The subscapularis muscle, the teres major muscle, the scapula, and latissimus dorsi, contribute to the posterior axillary border. The lateral border is delimited by the humerus. The axilla contains adipose tissue that surrounds and facilitates the movement of several critical structures during scapulothoracic motion. The main vessels passing through the axillary space are the axillary artery and vein with their branches. Important nerves passing through the same space are the brachial plexus and the long thoracic nerve.

### 3.2. Imaging

Axillary space is usually of great interest in breast cancer imaging [29,30]. Indeed, the axillary lymph node status is crucial for predicting recurrence and survival in breast cancer patients, making an accurate assessment of the region, important for staging. Sentinel LN biopsy (SNLB) has led to less invasive and more personalized axillary management, following evidence that suggested avoiding axillary lymph nodal dissection in selected node-positive and node-negative cases. Imaging techniques like axillary US, MRI, and US-guided biopsy play a key role in assessing nodal disease burden for treatment planning. Prediction models using imaging features are being developed to identify SLNB candidates. Collaboration between radiologists and surgeons is essential to optimize imaging’s role and improve treatment outcomes. Although US is the primary method for the evaluation of axillary nodes, breast MRI has advantages over US such as improved visualization of the axilla irrespective of patient body habitus and less operator dependence [31,32,33].

**Figure 2:**
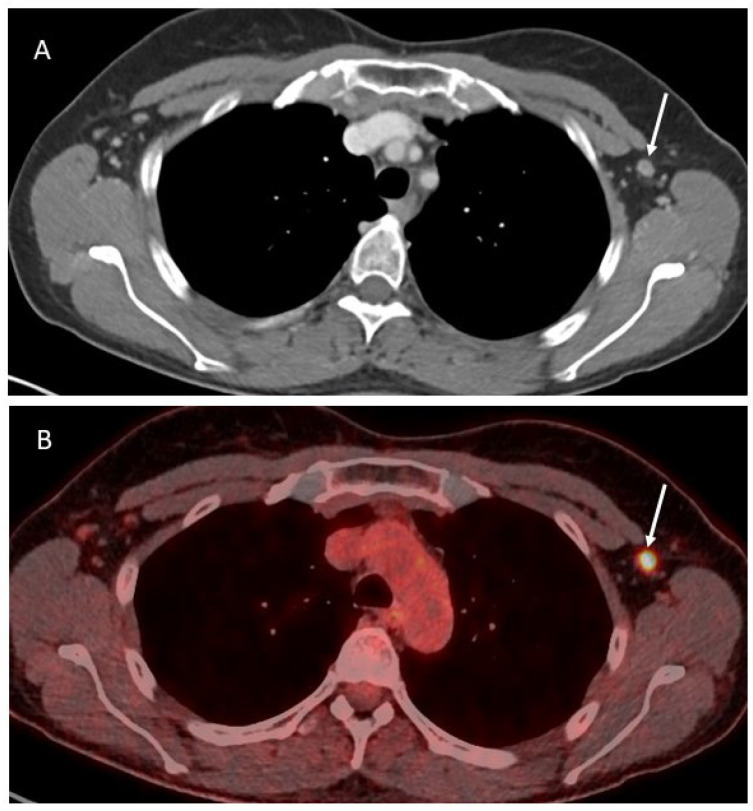
Axial contrast-enhanced CT image (**A**) showing a slightly enlarged lymph node in the left axilla (white arrow), corresponding to a pathologic activity on the PET-CT (**B**).

In OC patients, all the abovementioned imaging techniques are valid options to evaluate the presence of axillary lymphadenopathies (Figure 2).

However, considering the relatively superficial location of these lymph nodes, an adjunctive unexpensive and widely available imaging option is ultrasound (US). The criteria for pathological nodes at US include the size, as well as the shape and the internal structure. The size itself tends to be of limited value [34]. Therefore, morphological criteria may be additionally evaluated. The normal node has a thin hypoechogenic cortex in the periphery and an echogenic hilus. If there is a pronounced fatty part in the center, this becomes hypoechogenic. Pathological nodes tend to become more rounded, indicated as loss of the oval-shape. Therefore, when the normal length to width ratio >2 decreases to <1.5 the presence of metastasis can be suspected. The compression of the hilus, and especially the absence of the hilus, is highly suggestive of malignancy [35]. The use of color Doppler to differentiate benign form malignant lymph nodes is debated, with some authors suggesting good performances [36], and others showing fewer specific results [7]. The presence of axillary lymphadenopathies in OC patients does not preclude the possibility of an optimal debulking but it has to be carefully considered before planning the treatment.

Emerging imaging techniques are under investigation in the evaluation of lymph nodes. For instance, MR lymphography has demonstrated successful application in imaging and planning the treatment of conditions affecting the thoracic duct, lymphatic leaks, and other lymphatic abnormalities, but no evidence exists for the evaluation of axillary lymphadenopathies [37]. Diffusion weighted imaging (DWI) is a functional technique relying on the diffusion of water molecules in tissue to provide information about cellularity and tissue architecture [38]. The higher the cellularity of the tissue, the lower the diffusivity of free water molecules, the more restricted the diffusivity, which is typical for cancer tissues. The inclusion of functional MRI, specifically using DWI, has demonstrated superiority over the conventional MRI protocol in assessing lymph node status, both qualitatively and quantitatively [39]. A meta-analysis including 801 patients and 2305 lymph nodes demonstrated that DWI is a valuable method for differentiating between metastatic and nonmetastatic axillary lymph nodes [40]. When paired with the mean apparent diffusion coefficient value, DWI may enable a quantitative diagnosis of lymph node metastases. Large-scale, high-quality research can enhance the clinical relevance of diffusion-weighted imaging in distinguishing between metastatic and nonmetastatic axillary lymph nodes, providing crucial evidence for assessing the status of these lymph nodes [40]. Eventually, fine-needle aspiration biopsy (FNAB) may be needed to confirm that an image diagnosis of a metastatic lymph node is correct. FNAB should be performed under image control, and in this region, US is the best way to guide the biopsy.

### 3.3. Surgical Resection

The patient lies in a supine position, and the arm of the axilla to be biopsied is at 90 to 100 degrees of abduction. An oblique incision is performed by using the inferior axillary hair line as a landmark. The subcutaneous tissue is dissected to access the axillary fat pad after the incision of the clavipectoral fascia. It is recommended to visualize the axillary vein and start the dissection inferior to this. The gentle retraction of the pectoralis muscles allows a safe dissection of the level two lymph nodes, which can be easily accomplished by blunt dissection, thus preventing potential nerve damage by electrocautery. A small drain is recommended—depending on the depth of the dissection—to prevent or at least reduce postoperative seromas [41].

## 4. Mediastinum

### 4.1. Anatomy

**Figure 3:**
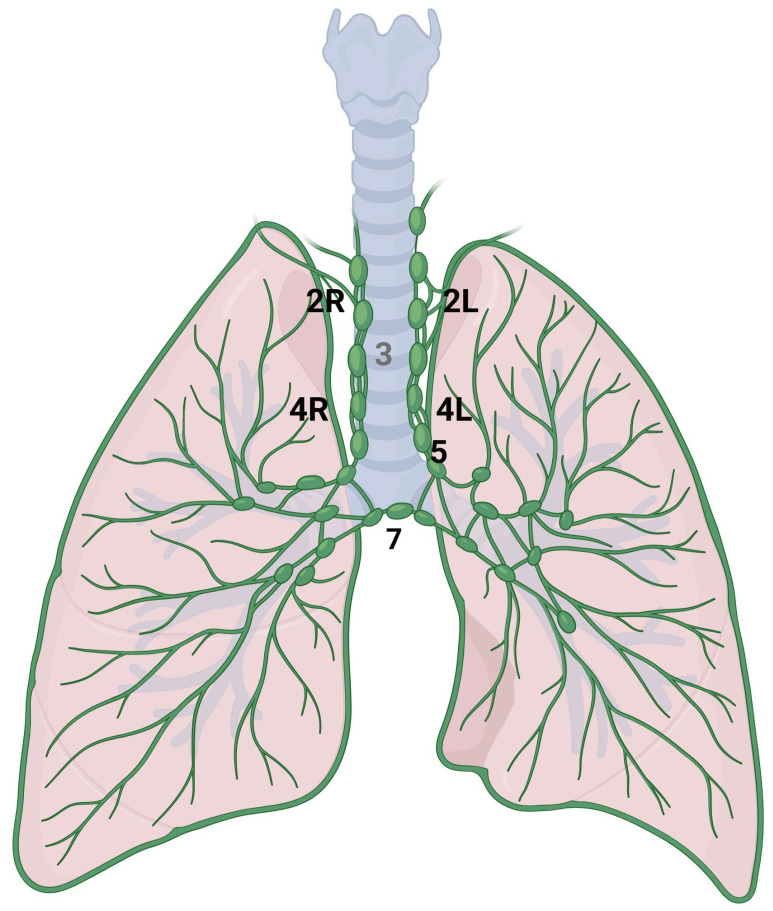
Anatomical representation of the following International Association for the Study of Lung Cancer (IASLC) mediastinal lymph node stations: right paratracheal (2R), left paratracheal (2L), lower right paratracheal (4R), lower left paratracheal (4L), subaortic (5) and subcarinal (7).

The mediastinum is located in the thoracic cavity, bordered by the sternum anteriorly, the vertebral column posteriorly, and laterally by the lungs. It is typically divided into four main compartments: the superior mediastinum, anterior mediastinum, middle mediastinum, and posterior mediastinum. The classification system, as defined by the International Association for the Study of Lung Cancer (IASLC), divides the mediastinal lymph node stations into the following categories (Figure 3) [42]. Station 2 comprises the upper paratracheal nodes that extend from the upper border of manubrium to the intersection of caudal margin of innominate vein with the trachea on the right (2R) and from the upper border of manubrium to the superior border of aortic arch on the left (2L). Station 3 comprises the pre-vascular and pre-vertebral nodes. Station 4 comprises the lower paratracheal nodes that extend from the intersection of the caudal margin of innominate vein with the trachea to the lower border of the azygos vein on the right (4R), and from the upper margin of the aortic arch to the upper rim of the left main pulmonary artery on the left (4L). Station 5 comprises the subaortic nodes (previously indicated as in the aorto-pulmonary space). Station 6 comprises the para-aortic nodes; station 7 comprises the subcarinal nodes [43].

### 4.2. Imaging

The current imaging guidelines from the European society of urogenital radiology (ESUR) recommend chest S-ray and abdominal CT for the pre-treatment evaluation of OC that can be extended to the chest in the case of pleural effusion; alternatively, other findings suggest the extra-abdominal diffusion of the disease [15]. However, these guidelines were published in 2010, and they are currently undergoing an update, which will include the chest CT. The criteria to consider a lymph node as suspicious for metastases are usually dimensional (short axis > 10 mm). The specificity of the sole short axis evaluation remains relatively low, and therefore, morphological and functional characteristics have been studied [17,19,44,45]. Accordingly, a meta-analysis showed that there was no significant difference between CT and MRI performance in correct interpretation of lymph nodes, whereas PET-CT showed significantly higher sensitivity and odds ratio compared to CT and MRI [25]. For this reason, when at pre-operative CT in OC, there are doubts about the presence of mediastinal lymphadenopathies, a PET-CT can be requested for confirmation and for choosing the nodes to be biopsied, if needed [46]. PET-CT findings of unexpected extra-abdominal lymph node metastases were reported in 15 of 95 patients with confirmed ovarian cancer [47]. Despite these promising results, PET-CT alone is not sensitive enough to replace surgical staging, but it is considered for detecting distant metastases that may contradict primary cytoreduction [48].

While PET imaging using 18F-labeled fluorodeoxyglucose is well-established in gynecologic cancers, several novel PET radiopharmaceuticals show promise for diagnosing, staging, and monitoring ovarian cancer [49]. One such example is the radiolabeled Fibroblast Activation Protein Inhibitor (FAPI). Dendl et al. evaluated the effects of 68Ga-FAPI PET-CT in 31 patients with gynecological malignancies and 14 patients with breast cancer, finding that 68Ga-FAPI PET exhibited high tracer uptake in both primary and metastatic lesions, with better tumor-to-background ratios compared to 18F-FDG PET-CT [50].

### 4.3. Surgical Resection

Radical mediastinal lymphadenectomy consists of the complete removal of all the lymphnodes of each nodal station and represents a standard surgical part of the operation when performing lung lobectomy for primary lung cancer [51]. On the other side, mediastinal nodal sampling consists of removal of part of one lymph node, or the removal of one lymph node of a selected nodal station or the removal of specific stations highly suspicious of metastatic involvement on the basis of preoperative imaging. On the right mediastinal side, stations 2 and 4 and 7, 8, and 9 are dissected; on the left side, stations 5 and 6 (aorto-pulmonary window and para-aortic) are completely removed [52]. In the case of mediastinal metastatic involvement from extra-pulmonary tumors, cito-histology confirmation is sufficient in most of cases, and bronchoscopic endobronchial ultrasound (EBUS) transbronchial needle biopsy (TBNA) is the gold standard [53]. If diagnostic nodal biopsy is required from districts not amenable of bronchoscopic exploration (paraortic, internal mammary chains) videothoracoscopy (VAT) or robotic assisted thoracoscopy (RAT) represent the best approaches; cervical mediastinoscopy—although effective—is not routinely used any more in the vast majority of centers.

## 5. Pericardiophrenic Fat

### 5.1. Anatomy

The pericardiophrenic lymph nodes are located in the fatty tissue surrounding the base of the heart in an extra-pleural and extra-abdominal space. They may be further divided into anterior, median/lateral, and posterior spaces. The anterior (retroxiphoid) cardiophrenic nodes collect lymph drainage from the anterior chest, supra-umbilical abdominal wall, anterior diaphragm, liver surface, and medial breast. The median or lateral (lateropericardial) cardiophrenic nodes drain lymph from intrathoracic organs, including the para-esophageal and median tracheobronchial chains. The posterior (juxta-esophageal) cardiophrenic nodes drain lymph from the chest wall, posterior pleura, esophagus, and posterior diaphragm ending in the left thoracic duct or right lymphatic duct.

### 5.2. Imaging

Cardiophrenic lymph nodes are usually so small that they are not detected in healthy subjects. Enlarged cardiophrenic lymph nodes are identified in 10.5% to 62% of patients with advanced epithelial ovarian cancer [54], with prevalence varying based on the radiological criteria used for defining pathological nodes. Indeed, there is no consensus on the optimal short-axis length of cardiophrenic lymph nodes that should be classified as pathological, with cut-off values ranging from 5 mm to 10 mm. The ESUR guidelines and node-RADS classification suggest a short axis >5 mm to consider a cardiophrenic lymph node as positive [15,17]. On the other hand, the RECIST 1.1 criteria do not consider a different criterion for this group of lymph nodes, sticking to the >10 mm short axis, the cut-off for malignancy [19]. Even international gynecological oncology guidelines do not specify the optimal imaging techniques for detecting pathological cardiophrenic lymph nodes in ovarian cancer, with consequent predictive capability for confirming metastatic disease ranging between 57% and 95%, based on the imaging modality utilized, the type of surgical intervention (primary vs. interval debulking), and the specific cut-off value applied for malignancy [54,55]. As previously mentioned, CT is the recommended imaging technique for pre-surgical evaluation and staging purposes [14,15]. MRI is mostly reserved for cases in which CT scanning is contraindicated, mainly because of costs, time, and need for specific expertise in the interpretation of MRI. In the evaluation of pericardiophrenic lymph nodes, PET-CT may detect higher glucose metabolism in a non-enlarged pathological lymph node. However, the European Association of Nuclear Medicine (EANM) states that the available evidence is limited, and no definitive conclusions can be drawn regarding the replacement of diagnostic CT by PET-CT in treatment planning [47].

### 5.3. Surgical Resection

Pericardiophrenic lymph node can be resected from the abdomen, the chest, or both [56]. In the case of the trans-abdominal approach, they are usually resected after diaphragmatic stripping with or without full thickness resection. The ventral aspect of the diaphragm is incised at the anterior medio-lateral edge of the centrum tendineum, taking care not to injure hepatic veins as well as phrenic vessels. The dissection is accomplished following the direction of diaphragmatic fibers, after the full mobilization of the liver caudally and posteriorly. A trans-diaphragmatic pleurotomy is then performed, thus entering the right pleural cavity: if the patient has been previously intubated by a double-lumen oro-tracheal tube, the right lung is easily excluded from the ventilation; in the case of a single-lumen tube, the anesthesiologist should provide a low-volume ventilation to allow a proper inspection and palpation of the pericardio-phrenic angle. Lymphadenectomy is then performed through the diaphragmatic incision; in the case of difficult exposure, video-assisted thoracoscopy (VAT) could be required. Chest drain is not mandatory, but it is recommended to prevent post-operative pneumothorax or pleural effusion. The diaphragm is then sutured without the need of any prosthetic reconstruction if no major diaphragmatic resection has been performed [57]. In the case of the trans-thoracic approach, VAT or robotic assisted thoracoscopy (RAT) approaches can be adopted, both requiring double-lumen oro-tracheal intubation to exclude the right lung form the ventilation. CO_2_ insufflation can be added to maximize lung collapse and further support tissue dissection, although it is not mandatory because a proper visualization and dissection can be obtained after standard lung deflation. Pleural cavity is inspected, including parietal pleural, pericardium, lung parenchyma, and the thoracic surface of the diaphragm; any required biopsy can be easily obtained by this approach.

## 6. Conclusions

OC is frequently diagnosed in advanced stages, and prognosis is related to residual disease following primary debulking surgery. The presence of extra-abdominal lymph nodes in the supraclavicular fossa, in the axilla, in the mediastinum, or in the pericardiophrenic fat indicates advanced-stage disease and may result in poorer prognosis for these patients. However, appropriate imaging evaluation and consideration for surgical resection, if deemed feasible in a multidisciplinary evaluation, may help to achieve an optimal cytoreduction even in advanced stages.

## Figures and Tables

**Table 1:** Thoracic sites of lymphadenopathies in ovarian cancer patients, with the most appropriate imaging modalities and surgical tips.

	US	CT	MRI	PET-CT	Imaging Tips	Surgical Resection Tips
**Supraclavicula fossa**		X		X	Refer to node-RADS [17] for CT imaging criteria of malignancy. PET-CT more accurate.	Traditional approached by scalene LN biopsy.
**Axilla**	X	X	X	X	Given the superficial position, US is the best imaging modality for assessment of these LN and to guide biopsies	Surgical procedure performed as for breast cancer.
**Mediastinum**		X		X	Refer to node-RADS [17] for CT imaging criteria of malignancy. PET-CT more accurate.	Consider EBUS-TBNA to make a differential diagnosis between metastatic and inflammatory enlarged LNs.
**Pericardiophrenic fat**		X	X	X	Refer to node-RADS [17] for CT and MRI criteria of malignancy. PET-CT more accurate.	Frequently approached through a trans-diaphragmatic approach from the abdomen. In case of difficult exposure, minimally invasive thoracic approaches can be adopted.

US = ultrasound; CT = computed tomography; MRI = magnetic resonance imaging; PET-CT = positron emission tomography–computed tomography.
